# Supplementary material for: Learning curve and surgical outcome of robotic assisted colorectal surgery with ERAS program
Source: Sci Rep. 2022 Nov 29;12:20566. doi: 10.1038/s41598-022-24665-w (PMC9709162; doi:10.1038/s41598-022-24665-w)
Supplement: Supplementary file 2 — Supplementary Information 2. [file 41598_2022_24665_MOESM2_ESM.zip › RAL Raw data-Ñ[▒K/131/OP N_ñw╝╨░O▒Kñσ.pdf]

W52 / W52

臺中榮民總醫院
手術室護理記錄

[redacted]

基本資料

- 室別： 開刀房-17室，手術科別： CRS，手術類別： 常規，日期： 2021/12/06
- 進入時間： 1825 ； 麻醉開始時間： 1850 ； Time Out時間： 2012 ； 劃刀時間： 2015 ； 手術結束時間： 0115 送出時間： 0140 ；
- 麻醉方式： GE
  診斷：Malignant tumor of rectum SP CCRT
  手術名稱：Robot Assisted Low Anterior Resection
  手術局部用藥：NIL
  術前預防性抗生素：
  - 抗生素名稱一： Veterin 1GM IVD/由麻醉護理師執行
  - 時間： 1950
  - 上次簽章: 張玉梅

  ，追加預防性抗生素：
  - 抗生素名稱一： Veterin 1GM IVD/由麻醉護理師執行
  - 時間： 2350
  - 上次簽章: 劉裴瑀
- 手術醫師： 蔣鋒帆，林陳彥，蔡睿璋
- 麻醉醫師： VS 蔡志忠
-
  - 刷手護士A ： 陳妍希，時間： 1825，刷手護士B ： 黃鈺雯，時間： 1825，接替刷手一： 劉裴瑀，時間： 1930，接替刷手二： 李芩瑜，時間： 2000
  - 流動護士A ： 張玉梅，時間： 1825，接替流動一： 劉裴瑀，時間： 2200，接替流動二： 林郁蓉，時間： 0100
- 更多接替及流動護士： 接替刷手三： 宋岱蓉，時間： 2300

皮膚狀況

A-V Shunt： 無
術前： 完整
記錄者： 上次簽章: 張玉梅
術後： 完整
記錄者： 林郁蓉

手術準備

手術臥式 單一手術臥式

-
  - 手術臥式： 截石臥式
  - 使用附件： 頭圈，側身架，其它： 海綿墊肩*2
  - 皮膚準備： Hibiscrub，2% Chlorhexidine
  - 電燒： 使用電燒: 電極片位置： 其它： 導電軟墊，記錄者： 上次簽章: 張玉梅

止血帶T： 無

植入物

- Endo Wrist Stapler 45-4.3 *2/rectum

標本送檢

- 細菌培養 無
- 細胞學 無
- 冰凍切片 無
- 病理標本
  組織名稱一：Rectum
  ,
  組織名稱二：Distal cut end

,
組織名稱三： IMA root

- 截肢 無

標本交付 標本： 3 袋，標本： 0 盒，標本： 0 瓶，細胞學： 0 管，Culture Tube： 0 管，被交付醫師： Fellow 林陳彥，

- 記錄者： 林郁蓉
- 時間： 0140

傷口術後敷料

傷口敷料： 傷口敷料： 無

- 請於圖上標示部位：

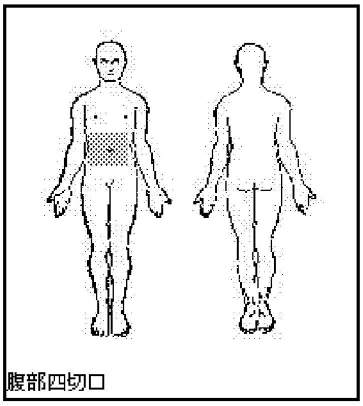

- 傷口敷料： Mepore

導管

導管	天數	導管資訊	插入日期	插入時間	更換日期	拔除日期	拔除時間	拔除原因	預計拔除/更換	預計拔除/更換日期
Foley(1)	2	號碼:16 Fr.; 部位:膀胱;	2021/12/06	1915						
Double Lumen CVC(1)	2	號碼:8Fr. ; 深度:15cm; 部位:jugular (右); 象限:RU;	2021/12/06	1920						
A-line (1)	2	號碼:20G ; 部位:右手;	2021/12/06	1935						

用物計數

- 手術中出血量： minimal c.c.
- 手術中尿量： 700 c.c.
-
  - 其它： 無

用物計數： 多切口同時縫合

- 1.器械： 正確，2.縫針： 正確，3.刀片： 正確，4.紗布： 正確，5.腹部墊： 未使用，6.紗球： 未使用，7.PEANUTS ： 未使用，8.紗條： 未使用，9.絲帶： 未使用，10.針頭： 未使用，11.Vessel loop： 未使用，12.Boots： 未使用，13.Jelco： 未使用，14.棉條： 未使用，
  - 第一次：
    - 刷手護士 ： 宋岱蓉
    - 流動護士： 上次簽章: 劉裴瑀
    - 時間： 2340

  ,
  - 第二次：
    - 刷手護士 ： 宋岱蓉
    - 流動護士： 上次簽章: 劉裴瑀
    - 時間： 2355

  ,
  - 第三次：
    - 刷手護士 ： 宋岱蓉
    - 流動護士： 上次簽章: 劉裴瑀
    - 時間： 0017

備註

備註： davinci 30度 XI scopy:No.2
davinci 0度 XI scopy:No.1

- 記錄者： 林郁蓉 上次簽章: 張玉梅
- 時間： 0140

填寫護理人員 林郁蓉

正式摘要

W52 / W52

doctorRank:B
